# Novel and Simplified Procedure to Test Immunity of Low-Power Voltage Transformers

**DOI:** 10.3390/s22155804

**Published:** 2022-08-03

**Authors:** Alessandro Mingotti, Lorenzo Peretto, Roberto Tinarelli

**Affiliations:** Department of Electrical, Electronic and Information Engineering, Guglielmo Marconi Alma Mater Studiorum, University of Bologna, Viale del Risorgimento 2, 40136 Bologna, Italy; lorenzo.peretto@unibo.it (L.P.); roberto.tinarelli3@unibo.it (R.T.)

**Keywords:** immunity, simplified, low-power instrument transformers, voltage transformers, accuracy, immunity, standard, voltage sensors

## Abstract

International technical committees put considerable efforts into the writing process of standards. They always try to find a tradeoff between the rigorous scientific requirements and the practical needs of manufacturers and final users. In addition, researchers keep investigating to improve the existing standards with new procedures, achievements, and findings. The purpose of this work is to contribute to that direction. It introduces a simplified and low-cost procedure to test low-power voltage transformers (LPVTs). The procedure is designed to assess the immunity of LPVTs when subjected to external electric fields. The need for this procedure comes from the existing immunity test, which is efficient but sometimes difficult to implement. The proposed one, instead, is simpler, cheaper, does not require the application of the rated voltage, and can be replicated at all voltage levels. In the paper, the procedure is described and demonstrated with experimental tests. From the results, it is possible to appreciate the validity of the proposed solution and the different ways it could be developed, implemented, and improved.

## 1. Introduction

Quite often, and in different fields, the average user exploits sensors and instrumentation as if they were perfect machines/devices. They completely rely on the obtained measurement results without questioning the goodness or the meaning of such results. Only when the measures are associated with billing or forensic purposes/issues are actions taken to ensure their trustworthiness.

However, among experts, the concern about the measurements’ validity is a daily activity. For example, system operators (SOs) are recently under a lot of pressure due to the changes occurring in the electrical grid. The European Commission foresaw that electrification is the key to decarbonization [1]. Such a process requires time, money, and several innovative solutions to become reality.

An example, which is already taking place, is the spread of renewable energy sources (RESs). Wind turbines, photovoltaic panels, hydroelectric solutions, etc., are being installed in the power network [2,3]. Note that, compared to traditional energy fuels, RESs are typically installed at all voltage levels, from low voltage (LV) to extra high voltage (EHV). Furthermore, their production is strictly associated with the source they use; hence, it cannot be predicted except for with the use of complex and not completely accurate forecast models [4,5].

Another current topic, which is a source of debate, is the cities’ infrastructure development for accepting the spread of electric vehicles (EVs). It is recognized that, once the main issues affecting them are solved, EVs may become the preferred solution for daily personal and industrial mobility. However, EV penetration will have a bifold impact, both on the electric grid and the civil infrastructure [6,7].

The given examples are just some of the challenges the grid is undergoing. However, they all lead to the conclusion that SOs must be ready to: (i) tackle all these challenges and (ii) include the incoming new assets in the daily management and control of the power network.

One of the key tools that was developed, and now is spreading among the electric grids, is the so-called low-power instrument transformer (LPIT). It is an innovative type of instrument transformer (IT) that produces very low-power outputs (few VAs), exploiting a variety of technologies. Such technologies and an overview of the relevant standards are given in Section 2.

LPITs, which could be voltage or current transformers (LPVTs and LPCTs, respectively), have new features that make them more appealing than legacy ITs. For example, they are smaller, more compact, and easier to install. Their increasing importance and relevance in the measurement field are demonstrated by the flourishing literature. For example, they are characterized vs. frequency and for power quality (PQ) applications in [8,9] and [10,11], respectively. Modeling is used in [12] for Rogowski coils, in [13] for an innovative current transformer (CT), and in [14] for legacy CTs. In [15], LPITs are characterized inside a realistic measurement chain containing phasor measurement units (PMUs).

In addition to the previous list of topics, this paper focuses on a further one: influence quantities. LPITs, but also all ITs in general, do not operate in nominal laboratory conditions. On the contrary, they are often installed in harsh environments in which they are subjected to a variety of influence quantities such as temperature, humidity, electromagnetic fields, pressure, etc. Consequently, such quantities are tackled in the literature. For example, the effect of temperature and how to treat/compensate is studied in [16,17,18]. Humidity has been recently studied by authors in [19]. As for the electromagnetic compatibility (EMC) issue, it is studied in [20] for electronic ITs. It is instead evaluated for automotive purposes in [21], whereas in [22] the authors study the EMC effects on ITs due to switching operations inside a substation.

This paper deals indeed with EMC issues, and more specifically, with the testing procedure to assess the effect of electric fields on LPVT accuracy. The test is prescribed by the standard IEC 61869-11 [23]; however, its applicability is sometimes difficult, in particular for high-voltage (HV) devices (Section 3 is dedicated to the description of the standard test). Therefore, considering the significance of the accuracy vs. immunity test, a new low-cost and simplified procedure is proposed and described. Afterward, the procedure is experimentally validated. Note that, according to the previously described literature and to the best knowledge of the authors, the studied topic has not been treated before, increasing the novelty of the paper.

All in all, the added value of this work can be summarized as follows. A simplified and low-cost procedure to test the immunity of LPVTs is described. Such a procedure is flexible enough to be implemented—with or without any modifications—to all types of LPVTs. Furthermore, it is suitable to be extended to all voltage levels, e.g., HV transformers, which the testing of is recognized to be more complicated than for the other voltage levels. Finally, the proposed procedure is not aimed at replacing the current standard. On the contrary, it demonstrates that new tests can be designed to simplify and improve the existing procedures.

The remaining sections are structured as follows. Section 2 provides a brief introduction to LPVTs and their working principle. The current immunity test, described in the standard, is the focus of Section 3. Section 4 describes in detail the proposed procedure and a way to test its validity. All the experimental results are collected and discussed in Section 5. Finally, the main achievements and the conclusion are summarized in Section 6, with emphasis on the potential practical benefits.

## 2. Low-Power Voltage Transformers

This section is dedicated to LPITs, focusing on LPVTs. Section 2.1 is a brief overview of the standards relevant to them, whereas Section 2.2 describes in a simple way the LPVT working principles.

### 2.1. IEC 61869 Series

ITs are standardized by the IEC 61869 series. It replaces the old IEC 60044 one. The new series comprises 15 documents. The most important are the IEC 61869-1 [24] and -6 [25] for ITs and LPITs, respectively, which contain the general prescriptions. Then, each type of transformer is standardized in a dedicated document. For example, LPVTs and LPCTs are described in [23] and [26], respectively. All in all, the IEC 61869 series is vivid, and its technical committee tries to keep it as updated as possible.

### 2.2. LPVT Working Principle

It is not possible to collect all LPVTs under the same working principle. However, thanks to the standard it is possible to provide a general description, and then detail each type of LPVT. First, from [25], an LPVT is defined as a “low-power instrument transformer for voltage measurement”, and an LPIT as an “arrangement, consisting of one or more current or voltage transformer(s) which may be connected to transmitting systems and secondary converters, all intended to transmit a low-power analogue or digital output signal to measuring instruments, meters and protective or control devices or similar apparatus”. Note how the low-power meaning has been intentionally kept generic to include a wide variety of devices (even if it is stated that the typical output is lower than 1 VA).

**Figure 1:**
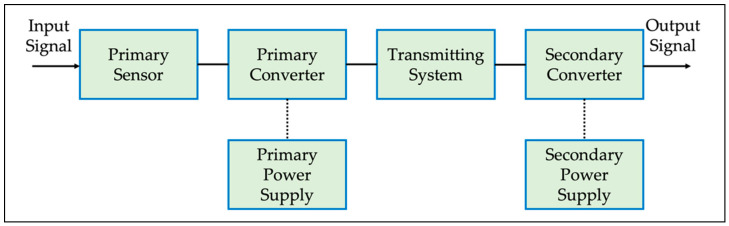
General block diagram of an LPIT. Rearranged from [25].

The general block diagram of an LPIT is depicted in Figure 1. It is generic enough to represent both LPCTs and LPVTs and all technologies implemented for their construction.

The key parts of the block diagram are the primary and secondary sensors/converters, and the possibility to feed the LPIT with an auxiliary supply.

Focusing on LPVTs, the voltage divider principle is the simplest and most adopted one. It uses passive components (two in the simplest version) to reduce the primary voltage to be measured. The voltage divider is typically implemented with resistors, capacitors [27,28], or a combination of both. Solutions with inductors are frequently not considered due to the resulting dimensions of the divider at power frequency (50 Hz, 60 Hz). The input/output expressions of the two types of voltage dividers are:

(1) $${\bar{V}}_{s}=\frac{R_{2}}{R_{1}+R_{2}}{\bar{V}}_{p},$$

(2) $${\bar{V}}_{s}=\frac{C_{1}}{C_{1}+C_{2}}{\bar{V}}_{p},$$

for the resistive and capacitive solutions, respectively. The expressions contain the primary ${\bar{V}}_{p}$ and secondary ${\bar{V}}_{s}$ voltage phasors, the primary $R_{1}$ and secondary $R_{2}$ resistors, and the primary $C_{1}$ and secondary $C_{2}$ capacitors.

The choice to decide which technology one should adopt depends on the application. For example, a capacitive divider (CD) does not suffer from heat dissipation due to resistors. Furthermore, in the ideal configuration, CDs are not frequency dependent. On the other hand, CDs cannot be used for DC applications such as resistive dividers (RDs). Finally, actual CDs always contain a resistive part in series to the capacitor (also known as an equivalent series resistor (ESR)), which compromises the ideal performance of the divider. Consequently, depending on the frequency content of the primary voltage, an RD may become the preferred solution.

## 3. Standard Test

Before detailing the proposed procedure, it is important to understand how the existing testing procedure works. This is the aim of this section.

### 3.1. Introduction and Setup

IEC 61869-11 dedicates an entire annex to the accuracy vs. immunity test. The reason for that is the assumption that adjacent phases in a three-phase system may influence each other’s accuracy. The standard defines that the test is valid for passive LPVTs for air-insulated systems, whereas the gas-insulated ones (GIS) shall be tested in their in-field configuration. No mention of active LPVTs is given.

**Figure 2:**
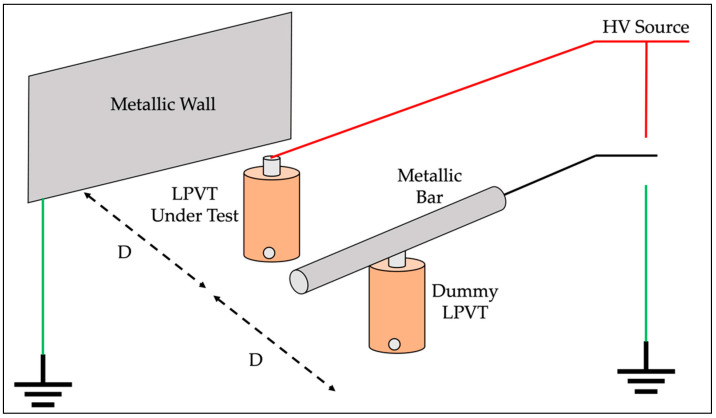
Simple structure of the accuracy vs. immunity test defined inside IEC 61869-11. Rearranged from [23].

The schematic of the setup given in and rearranged from [23] is shown in Figure 2.

The setup includes a metallic wall, a metallic bar, the LPVT under test, and a dummy LPVT. D is the real distance between adjacent LPVTs in a three-phase system. Consequently, the choice of D depends either on experience or on the final application (D is typically similar to the height of the LPVT). The same distance D is kept between the LPVT under test and the metallic wall. This wall has a length at least equal to D and a height of at least 1.5 the height of the LPVT. Finally, the metallic bar length is equal to D.

### 3.2. Standard Test Description

The test procedure described in [23] consists of two steps:

- Step 1. The LPVT under test is fed with its rated voltage, and the dummy LPVT—which is connected to the metallic bar—is grounded. The applied primary voltage and the secondary voltage of the LPVT under test are acquired to compute the actual transformation ratio and the phase displacement (referred to as $k_{1}$ and $∆\varphi_{1}$, respectively).
- Step 2. Both the LPVTs are fed with the rated primary voltage. Again, the secondary voltage of the LPVT under test and the primary voltage are acquired to compute the actual transformation ratio and the phase displacement (referred to as $k_{2}$ and $∆\varphi_{2}$, respectively).

Note that no output signal is collected from the dummy LPVT.

The test is then completed with a computational part. First, the difference between the two actual transformation ratios, divided by the one obtained in Step 2, is computed. This expression should be lower than 1/5 of the ratio error ε associated with the accuracy class (AC) of the LPVT under test. Second, the difference between the phase differences is computed. Such a value shall be lower than 1/3 of the phase displacement ∆φ associated with the AC of the LPVT under test.

To summarize:

(3) $$\frac{k_{2}-k_{1}}{k_{1}}100<\frac{1}{5}\varepsilon ,$$

(4) $$∆\varphi_{2}-∆\varphi_{1}<\frac{1}{3}∆\varphi ,$$

in which all symbols were previously introduced. Note, considering the terms inside (3) and (4), the associated measurement units are percentages and radians, respectively.

## 4. Proposed Procedure

### 4.1. Introduction

From Section 3 many observations can be drawn. For example, the described test setup may become difficult to implement for HV and EHV systems. The reasons are mainly three: (i) it is typically true that the higher the voltage, the higher the device will be, and hence, the limitations on the distances may become too strict; (ii) there is some additional equipment to complete the setup that may become too complex to obtain and install; and (iii) tests are performed at the rated voltage.

Overall, it would be preferable to have a simpler and more flexible test to easily verify the accuracy of an LPVT.

### 4.2. Simplified and Low-Cost Test Procedure

**Figure 3:**
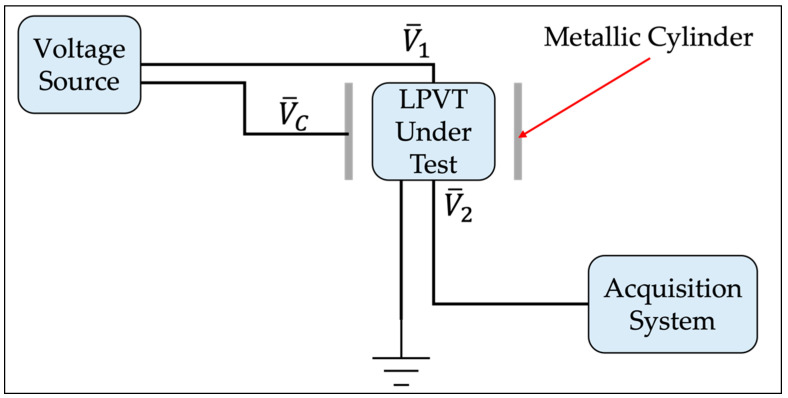
Schematic setup of the proposed test procedure.

This subsection presents, in detail, the experimental procedure to test the accuracy vs. immunity of LPVTs. The schematic setup of such a procedure is depicted in Figure 3.

The setup consists of just four components: the LPVT under test, a metallic cylinder, an LV voltage source, and an acquisition system. The procedure consists of applying an LV voltage ${\bar{V}}_{1}$ to the primary of the LPVT under tests, and a cylinder voltage ${\bar{V}}_{C}$ to the metallic cylinder which is positioned around the LPVT. Both voltages have a 60 Hz frequency. Afterwards, the secondary voltage ${\bar{V}}_{2}$ of the LPVT under test is acquired with a data acquisition system (with differential inputs, not grounded). A note on the frequency: its value was chosen because (i) it is a frequency simple to reproduce; (ii) 50 Hz would cause the beat phenomenon with the 50 Hz of the laboratory/building; and (iii) it is sufficient, for the test effectiveness, that ${\bar{V}}_{1}$ and ${\bar{V}}_{C}$ share the same frequency, no matter which one.

**Figure 4:**
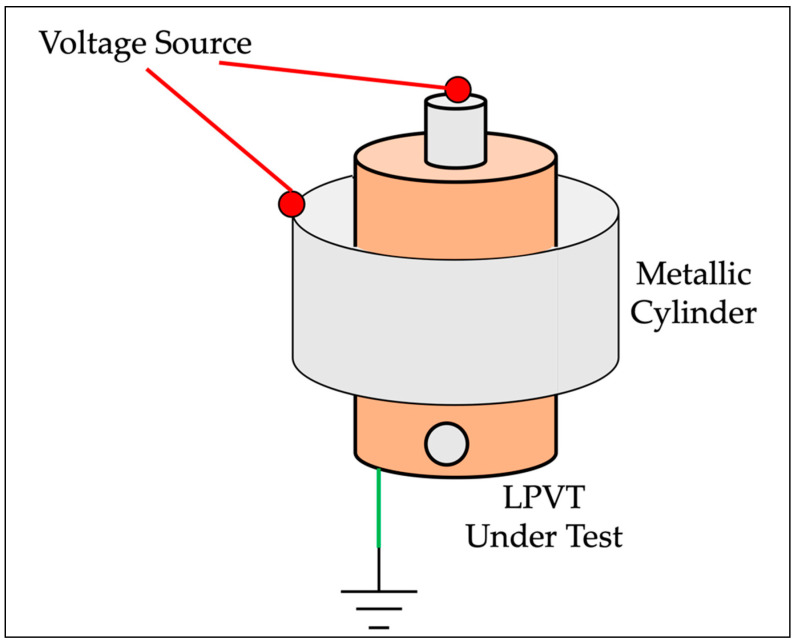
Positioning between the LPVT under test and the metallic cylinder.

To better understand the LPVT/cylinder reciprocal positioning, Figure 4 is introduced.

The requirements for the metallic cylinder are a centered position with respect to the LPVT and the homogeneity of its surface (i.e., it shall be “closed”). More comments are given after the presentation of the results.

The procedure ends with the ${\bar{V}}_{2}$ assessment depending on the ${\bar{V}}_{1}$ and ${\bar{V}}_{C}$ voltages.

### 4.3. Experimental Implementation of the Test Procedure

This section describes the proposed procedure which is then applied to off-the-shelf LPVTs. The test setup consists of:

- LPVTs. Four commercial LPVTs are tested. One of them does not include any shielding in its structure. This device is used as further proof of the test validity. The features of the four LPVTs are listed in Table 1. Note that the LPVTs are referred to as A, B, C, and D. A is passive and capacitive, B is active and capacitive, C is passive and resistive, and D, the unshielded, is passive and capacitive. All devices share the same 0.5 accuracy class. This class features a 0.5% limit on the ratio error and a 6 mrad limit on the phase displacement [23]. All LPVTs have an analogue output, which was collected with the data acquisition system.
- Voltage Source. The Fluke 6105A calibrator [29] is used to feed both the LPVT and the metallic cylinder. It is fundamental to mention that the device was not selected for its metrological performance. It was chosen because it is capable of providing two voltage outputs with the same frequency. That is the reason why no accuracy specifications are given here. As for the applied voltage amplitudes, a fixed 7 V amplitude is selected as ${\bar{V}}_{1}$, whereas the set {100, 200, 300, 400, 500, 600, 700, 800, 900, 1000} V is selected for ${\bar{V}}_{C}$.
- Metallic cylinder. A 30 cm-diameter aluminum cylinder is used for the tests.
- Data acquisition system (DAQ). A NI 9239 board is used to collect both ${\bar{V}}_{1}$ and ${\bar{V}}_{2}$. The features of the DAQ are collected in Table 2.

The test procedure in a nutshell: Each LPVT was fed with 7 V for the entire test duration. Then, each voltage from 100 V to 1000 was applied to the metallic cylinder, and 100 measurements of the LPVT secondary voltage ${\bar{V}}_{2}$ were collected to obtain a mean value and a standard deviation of the mean. The collected phasors resulted from a sampling window of ten periods of the fundamental signal and the application of the discrete Fourier transform. As for the phase-angle of the collected quantities, the difference between the phase-angle of ${\bar{V}}_{2}$ and ${\bar{V}}_{1}$ was used.

The 7 V choice for ${\bar{V}}_{1}$ was adopted because, after preliminary testing: (i) it is preferable to impose a potential to the primary terminals, instead of keeping it floating; (ii) grounding the primary terminal is another option, which is not realistic; and (iii) it is that low that it can be generated with whatever voltage source (even a function generator).

Note that several preliminary tests were performed. Such tests are not included in the results for the sake of clarity and significance. However, it was found that grounding the primary terminal results in similar results, but with an amplitude far lower than in the 7 V case. Furthermore, grounding the primary terminal results in the parallel between the primary and secondary impedances of the divider. Such a configuration is far from the actual one.

Another test was performed without any voltage on the metallic cylinder (${\bar{V}}_{C}=0$). Of course, no influence on the output of the LPVTs was found.

### 4.4. Validation of the Procedure

To understand whether the proposed procedure applies to realistic scenarios, a reference test is needed. Therefore, the standard procedure described in Section 3 was implemented. From the comparison of the two sets of results, the proposed procedure, which is a proof-of-concept, can be validated.

**Figure 5:**
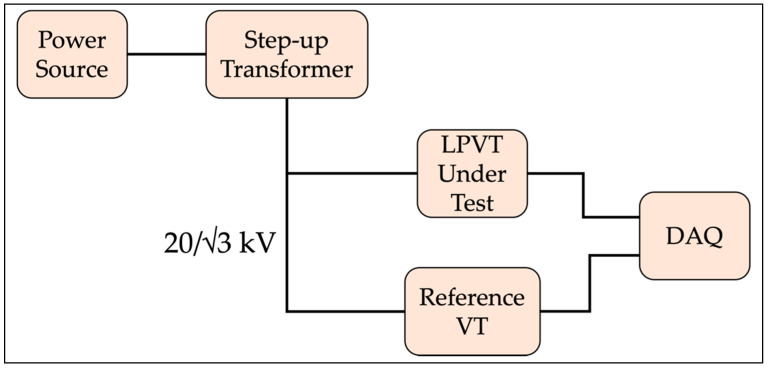
Simple schematic of the setup used to replicate the one in Figure 2.

Hence, a measurement setup to replicate the condition of Figure 2 is needed. Such a setup is illustrated in Figure 5. For the sake of the graph clarity, the additional required elements (metallic wall, bar, etc.) are not included.

It contains:

- An Agilent 6813B power source. It provides a stable 50 Hz voltage (less than 100 V) to the step-up transformer.
- A step-up transformer. It is used to increase the voltage to the LPVTs’ rated value. For all LPVTs, the rated voltage is 20/√3 kV (see Table 1).
- LPVTs under tests. These were already described.
- A reference voltage transformer (VT). To assess the output of the LPVTs, it is necessary to know the primary voltage applied to them. Hence, a capacitive reference divider with a 5981 ratio is adopted. It features an accuracy of three parts per thousand.
- A DAQ. The same previously described NI 9239 is used. The outputs of the LPVTs and the reference never exceed 10 V.

The setup was exploited to replicate the two-step test described in Section 3.2. For each step, and for each LPVT, 100 measurements of the actual ratio and the phase displacement were performed to obtain a mean value and its standard deviation of the mean.

## 5. Results

### 5.1. Results of the Proposed Procedure

**Figure 6:**
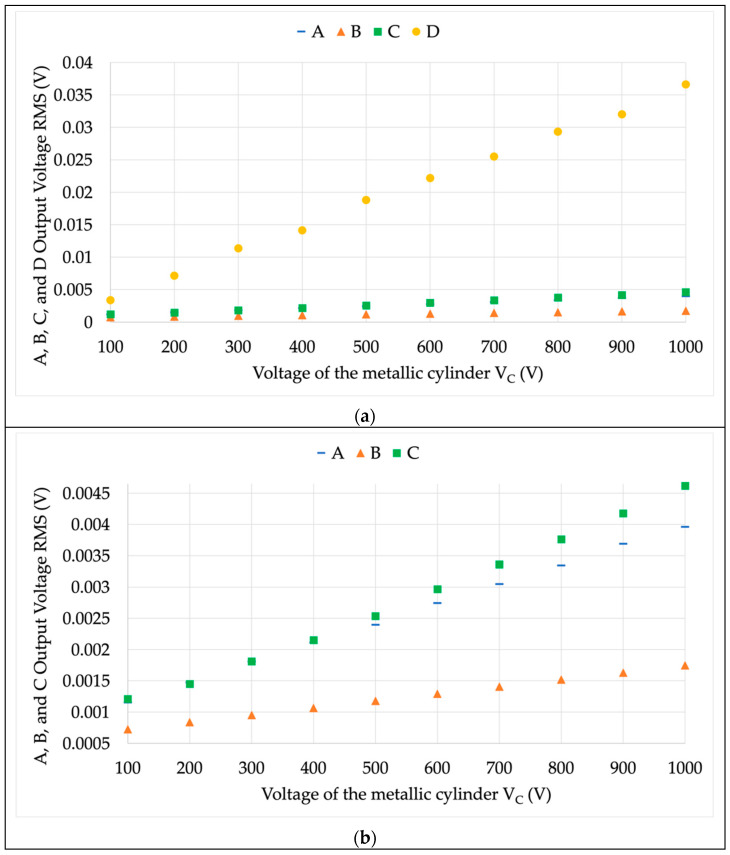
Secondary RMS of the voltages acquired from the LPVTs under test during the proposed procedure test. (**a**) Complete graph; (**b**) zoom in on A, B, and C.

**Figure 7:**
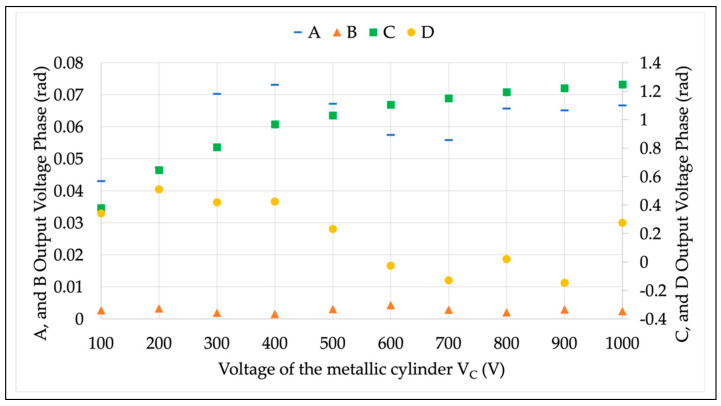
Secondary phase-angle of the voltages acquired from the LPVTs under test during the proposed procedure test.

The first set of results is described in Figure 6 and Figure 7. The former figure illustrates the RMS variation of the four LPVTs under test in the case of a varying ${\bar{V}}_{C}$. The latter figure, instead, contains the phase difference values with the same varying ${\bar{V}}_{C}$. In both figures, a color code for the LPVTs is used to increase the readability. Blue, orange, green, and yellow indicate LPVT A, B, C, and D, respectively. A note on the axes: to increase the readability, the C and D results are moved to the secondary vertical axis in Figure 7.

Looking at the results, starting from Figure 6, the linear contribution of ${\bar{V}}_{C}$ to the secondary voltage of the devices immediately emerges. Each device, which comes from a different manufacturer and was made with different technology, is differently affected by the external electric field. To better depict such behavior, Figure 6a plots the four results from the LPVTs, whereas Figure 6b does not contain LPVT D due to its higher values. Note that the D output voltage is at least one order of magnitude higher than the other tested devices.

Focusing on the results, the active LPVT (B) is significantly less affected compared to the resistive one (C), which is the most affected in the group. The behavior of B was expected considering that an active LPVT has a dedicated circuitry that adjusts and manipulates the device output. As seen from a comparison between the passive ones (Figure 6b), note that they have similar behavior for low values of ${\bar{V}}_{C}$. As soon as the voltage increases, instead, C is the most affected. That result is explained by the resistive nature of the device, which becomes resistive-capacitive when stray capacitances arise. It can be concluded that the resistive device is the most affected among the shielded ones.

To better quantify the differences, the values in the graph at 1000 V for A, B, C, and D are 1.744 mV, 3.959 mV, 4.61 mV, and 36.6 mV, respectively. The adopted number of digits is coherent with the standard deviation of the mean obtained from the experimental measurements. Such values are 10^−6^, 10^−6^, 10^−5^, and 10^−4^ for A, B, C, and D. The results also confirm the accuracy of the performed measurements and the detectable variations.

Overall, the first main result of the RMS test is that the proposed procedure allows for the detection of the different levels of sensitiveness of the LPVT under test, in terms of external electric fields.

Turning to Figure 7, almost all results have a different behavior compared to the RMS evaluation. The active device B is, again, almost not affected by the external electric field. That behavior is explained by the electronic circuit acting on the LPVT secondary output. However, as it was demonstrated above, the effects on the RMS voltage are evident. As for the resistive LPVT, C, it results in being the most affected by the electric field, even in this phase-angle evaluation. The obtained variations are in the order of 1 rad already at 500 V. Finally, for D, the unshielded device, it receives the impacting effect of the electric field, which results in a random variation of the phase angle associated with D’s secondary output.

A few notes on the results:

- The overall result is that the phase difference behavior is not linear, as in the case of the RMS.
- The applied voltage (${\bar{V}}_{1}$) is very limited in terms of magnitude; hence, the phase extraction is more complicated.
- As demonstrated by the literature (and in Section 5.2), most influence quantities affect the magnitude of the voltage. Consequently, the phase-angle evaluation becomes secondary, keeping the focus of the procedure on the voltage magnitude.
- The standard deviations associated with the phase-angle measurements are, for all cases, at least one order of magnitude lower than the measured quantity.

### 5.2. Results of the Test from the Standard

After the preliminary evaluation of the proposed procedure, the four selected LPVTs were subjected to the test described by the standard (see Section 3). The aim was to highlight the benefits and the limitations of the proposed procedure.

The results of the Step 1 and Step 2 tests, described in Section 3, are collected in Table 3. It contains, for each LPVT under test, the potential of the metallic bar (ground GND or HV), the test frequency, the measured transformation ratio k and its standard deviation of the mean $\sigma_{k}$, and the measured phase displacement φ and its standard deviation of the mean $\sigma_{\varphi }$.

From the values of $\sigma_{k}$ and $\sigma_{\varphi }$, the accuracy with which the associated parameters are evaluated can be appreciated. Such accuracy is coherent with the target uncertainty expected for k and φ.

Another note is on their absolute values. k values slightly differ from the ones in Table 1 because the latter values are the nominal ones. In other words, to determine the actual ratio, a characterization test is required (but out of the scope of this work). Analogously for φ, the measured ones differ from zero because correction quantities are summed/subtracted after the characterization process.

Once the results in Table 3 are obtained, the standard procedure prescribes to implement (3) and (4). The results are then compared with $\frac{1}{5}\varepsilon$ and $\frac{1}{3}∆\varphi$. In the case of 0.5 accuracy class devices, those limits correspond to 0.1% and 2 mrad.

The findings after the implementation of (3) and (4) are listed in Table 4. For the sake of the reader, (3) and (4) are used as titles in the table. Furthermore, the values in the table that exceed 0.1% and 2 mrad have a light orange background.

According to the figures, the unshielded LPVT significantly exceeds the defined limits for the ratio error and the phase displacement. In addition, the resistive C is slightly outside the phase limits. All the other figures lie within the limits, and it should be highlighted how the phase displacement of the two capacitive devices is completely insensitive to the external electric field.

### 5.3. Discussion

The whole set of results presented in Section 5.1 and Section 5.2 allows for a fruitful discussion on the proposed procedure and future perspectives.

**Figure 8:**
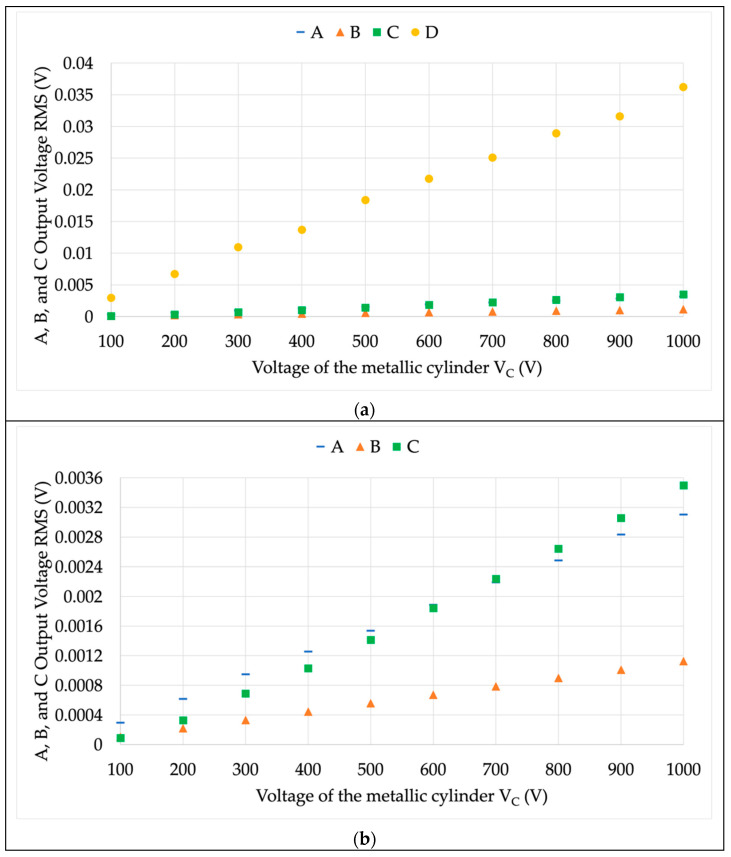
Secondary RMS of the LPVTs under test without the contribute of ${\bar{V}}_{1}$. (**a**) Complete graph; (**b**) zoom in on A, B, and C.

First, the standard test confirmed that the proposed procedure is capable of an effective immunity evaluation of an LPVT. Furthermore, the advantages of such a procedure are:

- The test uses LV for both the primary terminals (7 V) and the metallic cylinder (from 100 V to 1000 V). Consequently, no issues associated with the rated voltages apply, resulting in a clear benefit from the MV to the EHV. Note that using an MV may trigger other phenomena that vary the testing conditions. However, such conditions were completely changed with the procedure.
- There is no need for other (dummy) devices to run the test. In the standard procedure, the real configuration of the three-phase system must be replicated.
- The application of two iso-frequency voltages such as ${\bar{V}}_{1}$ and ${\bar{V}}_{C}$ allows for the removal of the ${\bar{V}}_{1}$ contribution from the results. One may comment that the obtained voltages are due to the primary voltage applied at the LPVT. Hence, the results presented in Figure 6 were replicated by removing the ${\bar{V}}_{1}$ = 7 V contribution. The results of this procedure are shown in Figure 8a,b. Note from the graphs how negligible the ${\bar{V}}_{1}$ contribution is, confirming the effectiveness of the procedure.
- It provides useful information on immunity for both the voltage magnitude and the phase-angle.
- The overall test is simpler, cheaper, and faster than the standard one. Such considerations include, for the duration, all the steps needed from the implementation of the measurement setup to the results’ assessment and evaluation. Furthermore, less instrumentation is needed.

At this point, it is useful to stress the future developments and the potential improvements of the method. In this way, it is possible to pave the way toward the implementation of the proposed procedure at a standardization level. The next steps can be summarized as follows:

- Harmonization of the metallic cylinder. In this study, a fixed metallic cylinder was used. This is because the dimensions of the LPVT under test are almost identical. However, at a commercial level, there is a variety of LPVTs with different dimensions. Hence, the metallic cylinder may become flexible to be adjusted to each LPVT. Furthermore, an interesting study may assess the metallic cylinder’s distance from the LPVT to find that which provides better results in terms of safety and immunity evaluation.
  Another aspect relevant to the metallic cylinder is its height. Again, each LPVT may have a different height, and this could result in different results during the immunity assessment.
- Type of LPVTs. The current standard prescribes that immunity tests shall be performed only on passive LPVTs. However, from the results, it was demonstrated that active LPVTs may also suffer from immunity. Therefore, a discussion on the types of LPVTs to be tested is needed. In addition, it was also shown that each working principle of an LPVT responds differently to the immunity test. This topic is also another source of discussion.
- Voltage level. The simplicity of the presented procedure allows for it being extended to all voltage levels. It is well known that HV and EHV tests are always critical and require dedicated laboratories and procedures. For this purpose, HV equipment will be tested to understand if the presented procedure may be replicated or if it needs some modifications.

## 6. Conclusions

Testing immunity is one of the critical tasks for manufacturers and users. It requires specific equipment and a strict procedure. However, tackling immunity is crucial because it affects the device’s accuracy. For this purpose, in this paper, a novel immunity test was presented and experimentally validated. The proposed test is cheaper, simpler, and faster than the one described in the relevant standard. Furthermore: (i) it is performed at a low voltage (no need for the rated voltage); and (ii) it does not require complicated equipment. The current test defined in the standard was used for a preliminary comparison and validation of the proposed one. From the presented results, as deeply described, it was demonstrated that the novel procedure allows for the immunity assessment of low-power voltage transformers. Therefore, the proposed procedure may contribute to the immunity testing of the LPITs and to the improvement of the existing tests. Finally, the pros and cons were discussed together with the future direction of the proposed procedure.

## Figures and Tables

**Table 1:** Characteristics of the four LPVTs under test.

Feature	A	B	C	D
Type of Divider	Capacitive	Capacitive	Resistive	Capacitive
Active/Passive	Passive	Active	Passive	Passive
Rated Primary Voltage (kV)	20/√3	20/√3	20/√3	20/√3
Rated Transformation Ratio (V/V)	8200	20,000/√3	6153	16,800
Accuracy Class (−)	0.5	0.5	0.5	0.5

**Table 2:** Features of the NI 9239 data acquisition board.

**Resolution**	24 bit	**Max Sampling Frequency**	50 kSa/s/ch
**Gain Error**	±0.03%	**Offset Error**	0.008%

**Table 3:** Results of Step 1 and Step 2 tests described in Section 3.

LPVT	Bar Potential	Frequency (Hz)	k (V/V)	$\sigma_{k}\;(V/V)$	φ	$\sigma_{\varphi }\;(\mathrm{rad})$
A	GND	60	8160.0720	7 × 10^−4^	0.0513622	1 × 10^−7^
	HV	60	8149.8043	8 × 10^−4^	0.0513455	2 × 10^−7^
B	GND	60	11,275.164	4 × 10^−3^	0.0023404	3 × 10^−7^
	HV	60	11,269.789	3 × 10^−3^	0.0023600	3 × 10^−7^
C	GND	60	6241.673	1 × 10^−3^	0.00697956	5 × 10^−7^
	HV	60	6241.371	1 × 10^−3^	0.004380	1 × 10^−6^
D	GND	60	16,884.7	9 × 10^−1^	0.06218	7 × 10^−5^
	HV	60	17,906	1	0.04788	7 × 10^−5^

**Table 4:** Results from the computation of (3) and (4).

LPVT	$\frac{k_{2}-k_{1}}{k_{1}}100\;(\%)$	$∆\varphi_{2}-∆\varphi_{1}\;(\mathrm{mrad})$
A	−0.126	−0.0168
B	−0.048	0.0196
C	−0.0048	−2.599
D	6.051	−14.3
